# Resident macrophages influence stem cell activity in the mammary gland

**DOI:** 10.1186/bcr2353

**Published:** 2009-08-26

**Authors:** David E Gyorki, Marie-Liesse Asselin-Labat, Nico van Rooijen, Geoffrey J Lindeman, Jane E Visvader

**Affiliations:** 1 VBCRC Laboratory, The Walter and Eliza Hall Institute of Medical Research, 1G Royal Parade, Parkville, Vic 3052, Australia; 2 Department of Medical Biology, The University of Melbourne, Parkville, Vic 3010, Australia; 3 Department of Molecular Cell Biology, Vrije Universiteit Medical Center, Van der Boechorstraat 7, 1081 BT, Amsterdam, The Netherlands; 4 Department of Medical Oncology, The Royal Melbourne Hospital, Grattan Street, Parkville, Vic 3050, Australia; 5 Department of Medicine, The University of Melbourne, Royal Melbourne Hospital, Parkville, Vic 3050, Australia

## Abstract

**Introduction:**

Macrophages in the mammary gland are essential for morphogenesis of the ductal epithelial tree and have been implicated in promoting breast tumor metastasis. Although it is well established that macrophages influence normal mammopoiesis, the mammary cell types that these accessory cells influence have not been determined. Here we have explored a role for macrophages in regulating mammary stem cell (MaSC) activity, by assessing the ability of MaSCs to reconstitute a mammary gland in a macrophage-depleted fat pad.

**Methods:**

Two different *in vivo *models were used to deplete macrophages from the mouse mammary fat pad, allowing us to examine the effect of macrophage deficiency on the mammary repopulating activity of MaSCs. Both the *Csf1*^*op*/*op *^mice and clodronate liposome-mediated ablation models entailed transplantation studies using the MaSC-enriched population.

**Results:**

We show that mammary repopulating ability is severely compromised when the wild-type MaSC-enriched subpopulation is transplanted into *Csf1*^*op*/*op *^fat pads. In reciprocal experiments, the MaSC-enriched subpopulation from *Csf1*^*op*/*op *^glands had reduced regenerative capacity in a wild-type environment. Utilizing an alternative strategy for selective depletion of macrophages from the mammary gland, we demonstrate that co-implantation of the MaSC-enriched subpopulation with clodronate-liposomes leads to a marked decrease in repopulating frequency and outgrowth potential.

**Conclusions:**

Our data reveal a key role for mammary gland macrophages in supporting stem/progenitor cell function and suggest that MaSCs require macrophage-derived factors to be fully functional. Macrophages may therefore constitute part of the mammary stem cell niche.

## Introduction

Increasing evidence suggests that macrophages play a role in the normal development of specific organs. In the pubertal mammary gland, macrophages are recruited to the highly mitotic terminal end buds (TEBs) from which ducts elongate and branch to give rise to a mature ductal tree. Macrophages are presumed to play a role in tissue remodeling by engulfing apoptotic epithelial cells during ductal morphogenesis [1,2]. Colony stimulating factor-1 (Csf1; also referred to as macrophage-Csf) is a key growth factor that regulates the proliferation and survival of macrophages [3]. *Csf1*^*op*/*op *^mice, homozygous for a null mutation in *Csf1*, exhibit multiple defects and have reduced macrophage numbers in most tissues including the mammary gland. These mice are severely runted, toothless and osteopetrotic, owing to an osteoclast deficiency that impairs bone resorption. The mammary glands of *Csf1*^*op*/*op *^mice display lower numbers of terminal end buds as well as reduced ductal branching and elongation [4]. During pregnancy, *Csf1*^*op*/*op *^glands develop precocious alveolar units but fail to switch to the lactational state resulting in impaired lactation [5]. Mammary-specific rescue has shown that the mammary gland phenotype is not due to secondary effects induced by a systemic Csf1 deficiency [6]. Targeted ablation of the receptor for *Csf1 *(*Csf1r *or *c-fms*), recapitulated all the phenotypes observed in *Csf1*^*op*/*op *^mice, although the receptor-null mice exhibited slightly more severe defects [7].

The nature of the mammary stem cell niche is yet to be defined but candidate cell types that may constitute the niche include epithelial cells, macrophages and eosinophils, among other cells in the mammary stroma (fibroblasts and adipocytes). One mechanism that may underlie the requirement of macrophages for normal mammopoiesis could be their interaction with mammary stem cells (MaSCs) [8,9]. To explore this possibility, we have utilized two *in vivo *approaches to determine whether macrophages are required for the function of MaSCs. We report that stem/progenitor cell activity is markedly attenuated by depletion of macrophages from the mammary gland, indicating that macrophages may influence stem cell function.

## Materials and methods

### Mouse strains

*Csf1*^*op*/*op *^mice were a kind gift from Dr Robin Anderson (Peter MacCallum Cancer Centre, Melbourne, Australia) and were on a pure BALB/c background. These mice were housed in a clean facility to optimise the viability of the homozygous mutant mice. Rosa26 mice were on a pure FVB/N background. All experiments were approved by the WEHI Animal Ethics Committee, and the care of animals was in accordance with institutional guidelines.

### Mammary stem cell preparation and cell culture

A single cell suspension of mammary cells was prepared from freshly harvested mammary glands and sorted by flow cytometry as previously described [8]. For transplantation assays, freshly harvested cells from the Lin^-^CD29^hi^CD24^+ ^fraction were transplanted in a volume of 10 μl into the de-epithelialized fourth mammary fat pads of three week-old syngeneic mice. Limiting dilution and statistical analyses were carried out as described by Shackleton et al [8]. For experiments using liposomes, the injection solution contained approximately 42 μg of clodronate liposomes per injection volume. Wholemount and histology analyses, as well as staining of mammary glands for LacZ activity using X-Gal (Sigma), were performed as described [8]. Clodronate liposomes were synthesized as previously described [10].

For *in vitro *cell culture assays, 100 freshly sorted epithelial cells (56 cells/cm^2^) were cultured for one week on 25,000 irradiated NIH-3T3 fibroblasts (14,000 cells/cm^2^) in 24-well culture dishes as described [8]. Colony size was measured using ImageJ [11].

## Results

**Figure 1:**
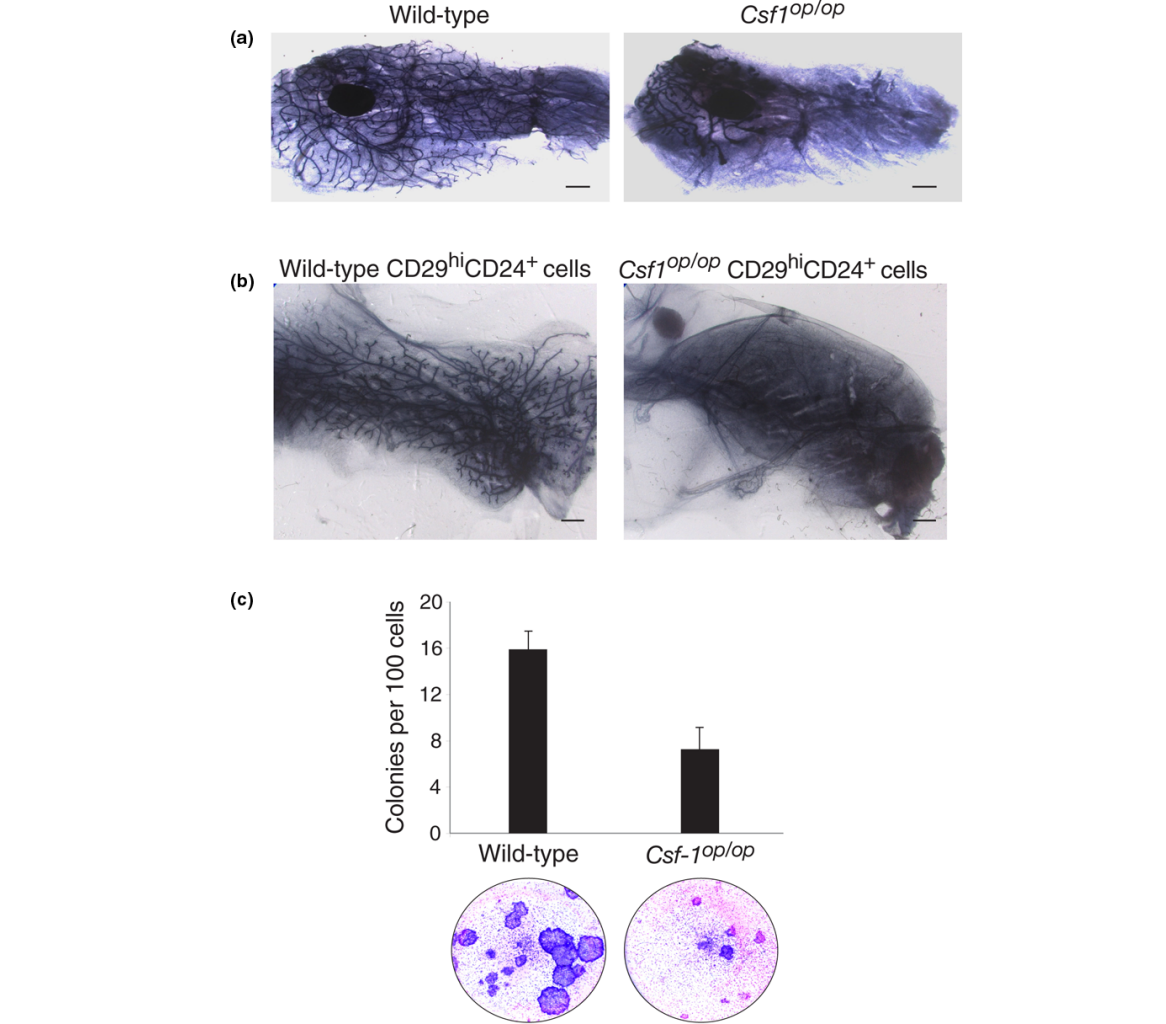
The MaSC-enriched population from *Csf1*^*op*/*op *^mice yields smaller outgrowths *in vivo *and has lower clonogenicity *in vitro*. **(a) **Wholemounts of fourth mammary glands from eight-week-old wild-type and *Csf1*^*op*/*op *^mice showing delayed filling of the fat pad and persistence of terminal end buds in mutant animals. **(b) **Wholemounts of outgrowths following transplantation of CD29^hi^CD24^+ ^cells from wild-type or *Csf1*^*op*/*op *^mammary glands into the cleared fat pads of syngeneic wild-type recipients. **(c) **CD29^hi^CD24^+ ^cells from *Csf1*^*op*/*op *^mammary glands exhibit lower clonogenicity and colony size than those from control glands. Histogram (upper panel) showing colony forming capacity of 100 CD29^hi^CD24^+ ^cells for each genotype. Error bars show standard error of the mean for three independent experiments. Lower panel shows cultures of CD29^hi^CD24^+ ^cells grown on fibroblast feeder layers. Scale bars = 1 mm. Csf1 = colony stimulating factor 1; MaSC = mammary stem cell.

To evaluate whether the impaired ductal morphogenesis that occurs in *Csf1*^*op*/*op *^mice (Figure 1a) reflects a perturbation in MaSC activity, freshly sorted CD29^hi^CD24^+ ^mammary epithelial cells were isolated from BALB/c *Csf1*^*op*/*op *^and wild-type glands. They were then transplanted into the de-epithelialized fat pads of three-week-old BALB/c mice at limiting dilution. The CD29^hi^CD24^+ ^subpopulation has previously been shown to be enriched for MaSCs but also contains mature myoepithelial cells and possibly basal progenitor cells [8]. Cells were purified from 12 week-old *Csf1*^*op*/*op *^mice and eight-week-old wild-type littermates, in order to compensate for the recognised delay in filling of the fat pad that occurs in *Csf1*^*op*/*op *^mutant mice [4]. The glands were harvested for wholemount analysis eight weeks post-transplantation.

Limiting dilution analysis of four independent experiments revealed that the stem cell frequency was reduced in *Csf1*^*op*/*op *^mice. The mammary repopulating frequency was 1 in 261 (95% confidence interval (CI) = 1/166 to 1/408) versus 1 in 97 (95% CI = 1/66 to 1/141) for *Csf1*^*op*/*op *^and wild-type mammary glands, respectively (*P *< 0.001; Table 1). Estimation of the absolute number of mammary repopulating cells revealed an 18-fold decrease in mutant glands (mean of 702 versus 40 cells per gland in wild-type and *Csf1*^*op*/*op*^mice, respectively). In addition to the decrease in repopulating frequency by CD29^hi^CD24^+ ^cells in the *Csf1*^*op*/*op *^group, only 5 of the 45 transplanted fat pads (11 ± 4%; mean ± standard error of the mean (SEM)) yielded outgrowths filling more than 50% of the fat pad, whereas 22 of the 45 transplanted fat pads (49 ± 4%) were filled by outgrowths from wild-type stem cells. Thus, despite being transplanted into a normal microenvironment, the MaSC-enriched population from *Csf1*^*op*/*op *^mice did not give rise to fully developed mammary outgrowths (Figure 1b), indicating that the function of mammary stem/progenitor cells had been attenuated in the macrophage-depleted environment.

**Table 1:** Limiting dilution analysis of wild-type or *Csf1*^*op*/*op *^CD29^hi^CD24^+ ^cells transplanted into cleared mammary fat pads

**Number of CD24^+^CD29^hi ^cells injected per fat pad**	Number of positive outgrowths*	
	**Wild-type**	***Csf1* ^*op*/*op*^**
60	3/4	0/4
100	7/11	5/11
120	2/4	1/4
200	20/22	12/22
240	3/4	2/4

**Repopulating frequency** (95% confidence interval)	**1/97** (1/66 to 1/141)	**1/261** (1/166 to 1/408)

Limiting dilution analysis of the repopulating frequency of mammary stem cell (MaSC)-enriched cells from either wild-type or *Csf1*^*op*/*op *^glands transplanted into wild-type recipients. Cells were injected into the cleared mammary fat pads of three-week-old syngeneic recipients. Results are pooled from four independent experiments (*P *= 0.00065). *Shown as number of outgrowths per number of injected fat pads. Csf1 = colony stimulating factor 1.

Consistent with the transplantation data, *in vitro *colony forming assays on fibroblast feeder layers revealed a two-fold reduction in the clonogenic potential of CD29^hi^CD24^+ ^cells from *Csf1*^*op*/*op *^mice relative to those from control mice (Figure 1c). The absolute numbers of clonogenic cells in the MaSC-enriched population per mammary gland from either wild-type or *Csf1*^*op*/*op*^mice were estimated to be 14,666 ± 1530 and 1306 ± 348, respectively (mean ± SEM, n = 3). Notably, a 2.4-fold decrease in colony size was also apparent on comparison of colonies derived from *Csf1*^*op*/*op *^CD29^hi^CD24^+ ^cells relative to those from wild-type mice. The addition of F4/80-positive mouse mammary macrophages to the clonogenic assays had no discernible effect on colony size or morphology, but may reflect inadequate culture conditions for macrophages in the epithelial cell assays (data not shown).

**Figure 2:**
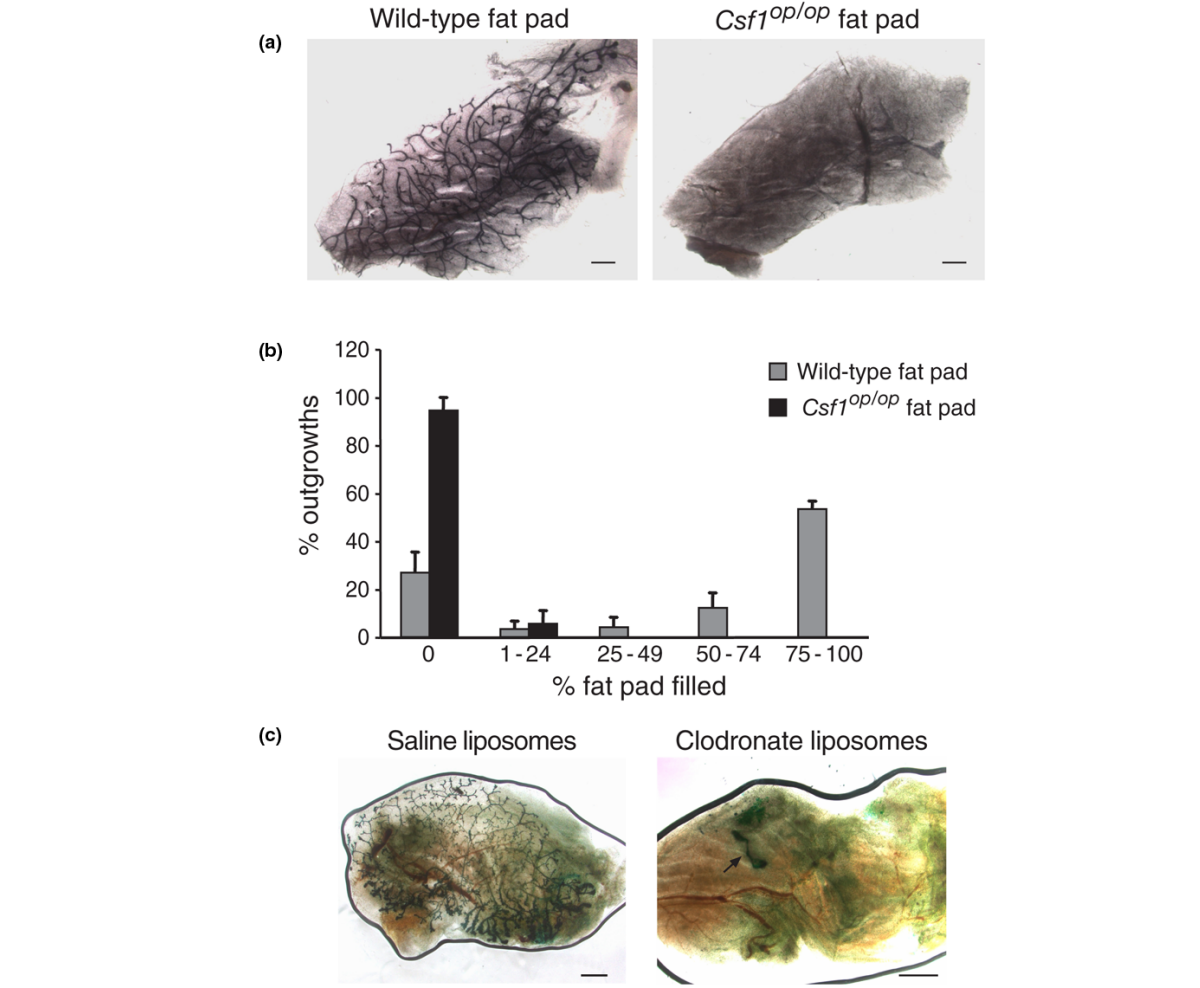
Wild-type mammary stem cells are unable to efficiently repopulate macrophage-deficient fat pads. **(a) **Wild-type CD29^hi^CD24^+ ^cells transplanted into *Csf1*^*op*/*op *^fat pads resulted in only a single small outgrowth whereas those transplanted into wild-type recipients resulted in extensive filling of the fat pad, all harvested at eight weeks post-transplantation. **(b) **Histogram showing percent outgrowth and the extent of fat pad filling following transplantation of normal CD29^hi^CD24^+ ^cells into wild-type or *Csf1*^*op*/*op *^fat pads. Error bars represent standard error of the mean for three independent experiments. **(c) **Clodronate liposomes inhibit mammary stem cell function. CD29^hi^CD24^+ ^cells (200) from Rosa26 mice were co-transplanted with liposomes containing either saline or clodronate into the cleared mammary fat pads of syngeneic wild-type female mice. The cells co-transplanted with clodronate liposomes resulted in no outgrowths or rudimentary structures, an example of which is shown. Scale bars = 1 mm. Csf1 = colony stimulating factor 1.

To investigate a potential role for macrophages in the mammary stem cell microenvironment, the wild-type MaSC-enriched population was next transplanted into the macrophage-deficient fat pads of *Csf1*^*op*/*op *^mice. Two hundred CD29^hi^CD24^+ ^cells isolated from the mammary glands of young wild-type female mice (BALB/c) were transplanted into either three-week-old wild-type or six-week-old *Csf1*^*op*/*op *^fat pads that had been cleared of endogenous epithelium. Older mutant recipient mice were used to compensate for the marked developmental delay that occurs in *Csf1*^*op*/*op *^mice. Substantial outgrowths were observed in wild-type recipients (18 of 24), whereas only a single small outgrowth (1 of 18) comprising two ducts was evident in *Csf1*^*op*/*op *^recipients (*P *< 0.00001; Figures 2a, b). These data suggest that mammary repopulating cells require the presence of resident macrophages in the mouse mammary gland.

To further investigate the effect of macrophage depletion from the mammary fat pad on stem cell activity, an alternative model of macrophage deficiency was sought. Clodronate is a bisphosphonate that can be delivered in a targeted manner when packaged into liposomes [10]. The liposomes are non-toxic until ingestion by macrophages, in which they are broken down by liposomal phospholipases to reveal the drug. Sufficient clodronate uptake by macrophages causes cell death by apoptosis. In co-transplantation experiments, CD29^hi^CD24^+ ^cells (200 cells) from the mammary glands of Rosa26 mice were coinjected into the cleared mammary fat pads with either clodronate-containing or saline-containing (control) liposomes. Six weeks after transplantation, the glands were harvested and the outgrowths analysed by staining for LacZ activity (see Materials and methods). Three independent experiments revealed outgrowths in 22 of 28 (77 ± 5%) fat pads coinjected with saline liposomes compared with only 4 of 28 (18 ± 10%) fat pads co-injected with clodronate-containing liposomes (*P *< 0.000001). Of the four outgrowths evident in the clodronate group, only one branching outgrowth was observed, while the remaining three were very rudimentary structures (Figure 2c).

## Discussion

Our data reveal that mammary macrophages contribute to normal stem/progenitor cell function in the developing mouse mammary gland. In a macrophage-deficient environment, the MaSC-enriched subpopulation had a markedly diminished capacity to reconstitute mammary outgrowths. The role of macrophages in this organ, however, appears to be supportive rather than integral to MaSC function, because these mice harbor some MaSCs and limited development can occur in *Csf1*^*op*/*op *^mice. Interestingly, even in a macrophage-replete system, the function of MaSCs from *Csf1*^*op*/*op *^glands was compromised. Indeed, both the absolute number and repopulating capability of MaSCs appear to have been altered by the macrophage-deficient environment, possibly reflecting downregulation of growth factor-mediated signal transduction pathways required for stem cell function.

Here we demonstrate the efficacy of clodronate-containing liposomes for macrophage depletion in the mammary gland. Clodronate liposomes have been successfully utilized for macrophage depletion from liver, spleen, lung, lymph nodes, joints, peritoneal cavity, and testis [10]. Local depletion of resident macrophages from the developing mammary gland by clodronate liposomes led to a marked decrease in repopulation by the MaSC-enriched fraction. The depletion of macrophages is likely to be temporary as the liposomes are phagocytosed within the first few days and macrophages presumably return to normal levels by the time of tissue harvest at six weeks post-transplantation. Therefore, mammary macrophages appear to be required for the early stages of repopulation by MaSCs and their progeny.

It is tempting to speculate that mammary macrophages constitute part of the normal MaSC microenvironment and facilitate the maintenance and/or proliferation of stem cells. Gene expression analysis has shown that the MaSC-enriched subpopulation expresses significantly higher levels of *Csf1 *(approximately five-fold by quantitiative RT-PCR) relative to the luminal epithelial subset. Only macrophages in the mammary gland appear to express the receptor (c-fms or Csf1R) for the Csf1 ligand under normal physiologic conditions, based on immunostaining for the receptor [4] as well as expression in *c-fms *reporter mice (data not shown). Csf1 may therefore be a chemoattractant for macrophages to the niche in which MaSCs are thought to reside in a basal location. Consistent with this notion, macrophages can be found around the cap cell region of TEBs [4], which has been proposed to be enriched for stem cells [12]. The factors released by macrophages that support stem cell activity are yet to be determined but may include macrophage-secreted cytokines such as CXCL12. Evidence for the direct participation of macrophages in the MaSC niche should eventually be possible through the prospective identification of a highly specific MaSC marker. The finding that the colonic niche was located at the base of the crypts allowed the localization of macrophages to this region, while electron microscopy identified a direct physical relation between colonic epithelial progenitor cells and macrophages [13].

## Conclusions

Our data demonstrate that depletion of mammary gland macrophages either by chemical ablation or the use of *Csf-1*^*op*/*op *^mutant mice alters mammary stem/progenitor cell activity, reflected in a substantially reduced repopulating frequency. The diminished outgrowth potential indicates a continued requirement for normal macrophages during ductal morphogenesis and may be mediated by effects on the putative basal progenitor cell. Overall, these findings indicate that macrophages play a role in supporting normal mammary stem cell function *in vivo *and suggest that they may constitute part of the normal MaSC microenvironment.

## Abbreviations

CI: confidence interval; CSF1: colony stimulating factor 1; MaSC: mammary stem cell; RT-PCR: reverse transcription polymerase chain reaction; SEM: standard error of the mean; TEB: terminal endbud.

## Competing interests

The authors declare that they have no competing interests.

## Authors' contributions

DG contributed to the conception and design, collection and assembly of data, data analysis and interpretation, manuscript writing. MA contributed to the collection and assembly of data. NvR provided the liposomal reagents. GJL contributed to the study conception and design, provision of study materials, data analysis and interpretation. JEV contributed to the conception and design, provision of study materials, data analysis and interpretation and manuscript writing. All authors read and approved the final manuscript.
